# Rapid progressive long esophageal stricture caused by gastroesophageal reflux disease after pylorus-preserving pancreatoduodenectomy

**DOI:** 10.1186/s12893-016-0137-2

**Published:** 2016-04-18

**Authors:** Masahide Fukaya, Tetsuya Abe, Masato Nagino

**Affiliations:** Division of Surgical Oncology, Department of Surgery, Nagoya University Graduate School of Medicine, 65 Tsurumai-cho Showa-ku, Nagoya, 466-8550 Japan

**Keywords:** Esophageal stricture, Esophagectomy, Pancreatoduodenectomy, Delayed gastric emptying

## Abstract

**Background:**

Delayed gastric emptying (DGE) is a major postoperative complication after pylorus-preserving pancreatoduodenectomy (PpPD) and sometimes causes reflux esophagitis. In most cases, this morbidity is controllable by proton-pump inhibitor (PPI) and very rarely results in esophageal stricture. Balloon dilation is usually performed for benign esophageal stricture, and esophagectomy was rarely elected. In the present case, there were two important problems of surgical procedure; how to perform esophageal reconstruction after PpPD and whether to preserve the stomach or not.

**Case presentation:**

A 63-year-old man underwent PpPD and Child reconstruction with Braun anastomosis for lower bile duct carcinoma. Two weeks after surgery DGE occurred, and a 10 cm long stricture from middle esophagus to cardia developed one and a half month after surgery in spite of the administration of antacids. Balloon dilation was performed, but perforation occurred. It was recovered with conservative treatment. Even the administration of a proton pump inhibitor (PPI) for approximately five mouths did not improve esophageal stricture. Simultaneous 24-h pH and bilirubin monitoring confirmed that this patient was resistant to PPI. We performed middle-lower esophagectomy with total gastrectomy to prevent gastric acid from injuring reconstructed organ and remnant esophagus through a right thoracoabdominal incision, and we also performed reconstruction with transverse colon, adding Roux-Y anastomosis, to prevent bile reflux to the remnant esophagus. Minor leakage developed during the postoperative course but was soon cured by conservative treatment. The patient started oral intake on the 25th postoperative day (POD) and was discharged on the 34th POD in good condition.

**Conclusion:**

Long esophageal stricture after PpPD was successfully treated by middle-lower esophagectomy and total gastrectomy with transverse colon reconstruction through a right thoracoabdominal incision. Conventional PD or SSPPD with Roux-en Y reconstruction rather than PpPD should be selected to reduce the risk of DGE and prevent bile reflux, in performing PD for patients with hiatal hernia or rapid metabolizer CYP2C19 genotype; otherwise, fundoplication such as Nissen and Toupet should be added.

## Background

Delayed gastric emptying (DGE) is a major postoperative complication after pylorus-preserving pancreatoduodenectomy (PpPD) and sometimes causes reflux esophagitis [1, 2]. In most cases, this morbidity is controllable by proton-pump inhibitor (PPI) and very rarely results in esophageal stricture. Conservative therapy, such as balloon dilation and the temporary placement of a self-expanding plastic stent, is usually performed for benign esophageal stricture, and surgical treatment was rarely elected [3]. Moreover, there have so far been few such reports on esophageal reconstruction after pancreatoduodenectomy. We report a patient with rapid progressive long esophageal stricture caused by gastroesophageal reflux disease (GERD) after PpPD in whom balloon dilation failed and subsequent esophagectomy with colon reconstruction was required.

## Case presentation

A 63-year-old man underwent PpPD and Child reconstruction with Braun anastomosis for lower bile duct carcinoma at another hospital. Two weeks after surgery, he vomited several times due to DGE, and a nasogastric tube was inserted into the stomach. On the 32nd POD, however, DGE improved and the nasogastric tube was removed, as dysphagia persisted. On the 41st POD, gastrointestinal endoscopy was performed, revealing stricture of the middle esophagus. PPI did not improve esophageal stricture; the patient then underwent balloon dilation on the 70th POD, but the esophagus was perforated. The esophagus recovered with fasting, antibiotics, and PPI without surgical treatment. As the stricture still remained, he was referred to our hospital for further treatment on the148th POD.

**Fig. 1:**
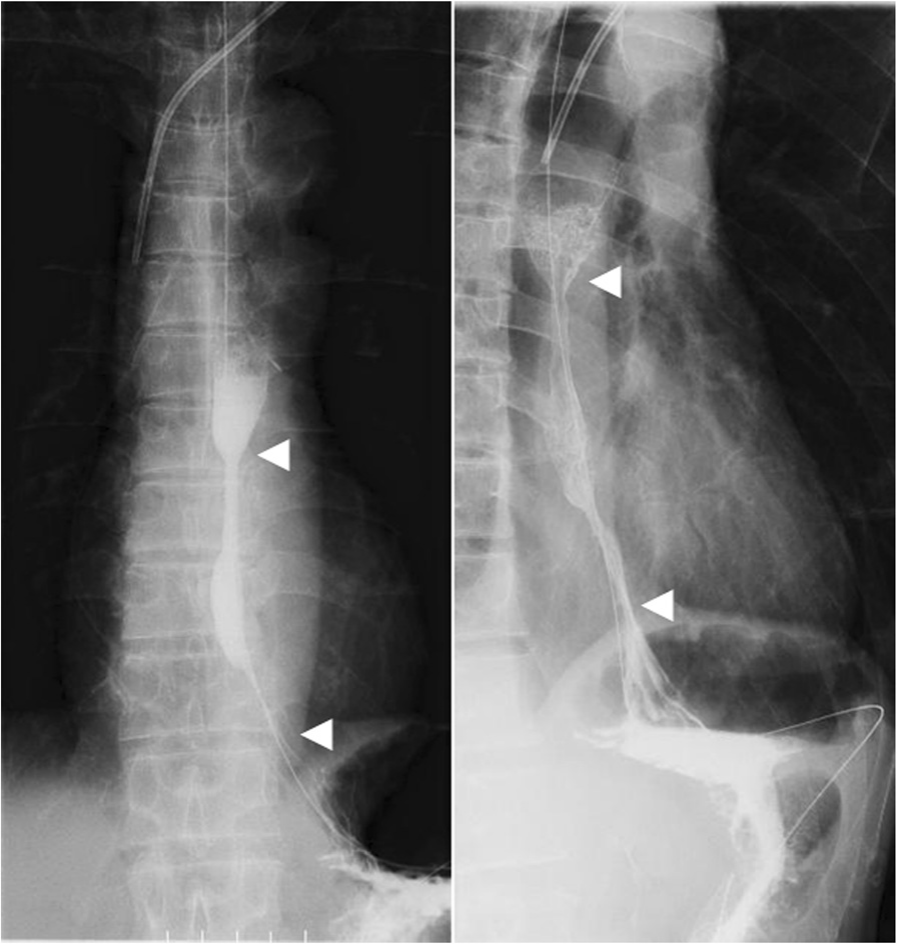
Upper gastrointestinal series. Severe long esophageal strictures and sliding esophageal hiatal hernia were confirmed. The narrow lesion is between *white arrows*; the length was 10 cm

**Fig. 2:**
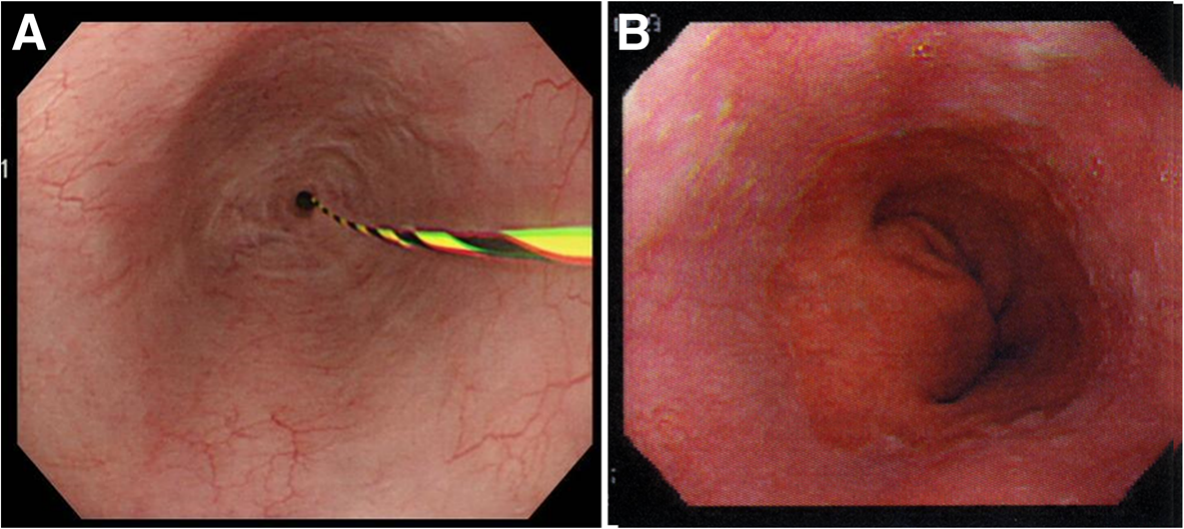
**a**. Endoscopic findings on the 150th POD after PpPD showed severe stricture of the middle thoracic esophagus and longitudinal esophageal ulcer scars on the oral side. **b** Endoscopic findings before PpPD showed sliding esophageal hiatal hernia and mild esophagitis, which was classified as Grade A according to the Los Angeles classification

**Fig. 3:**
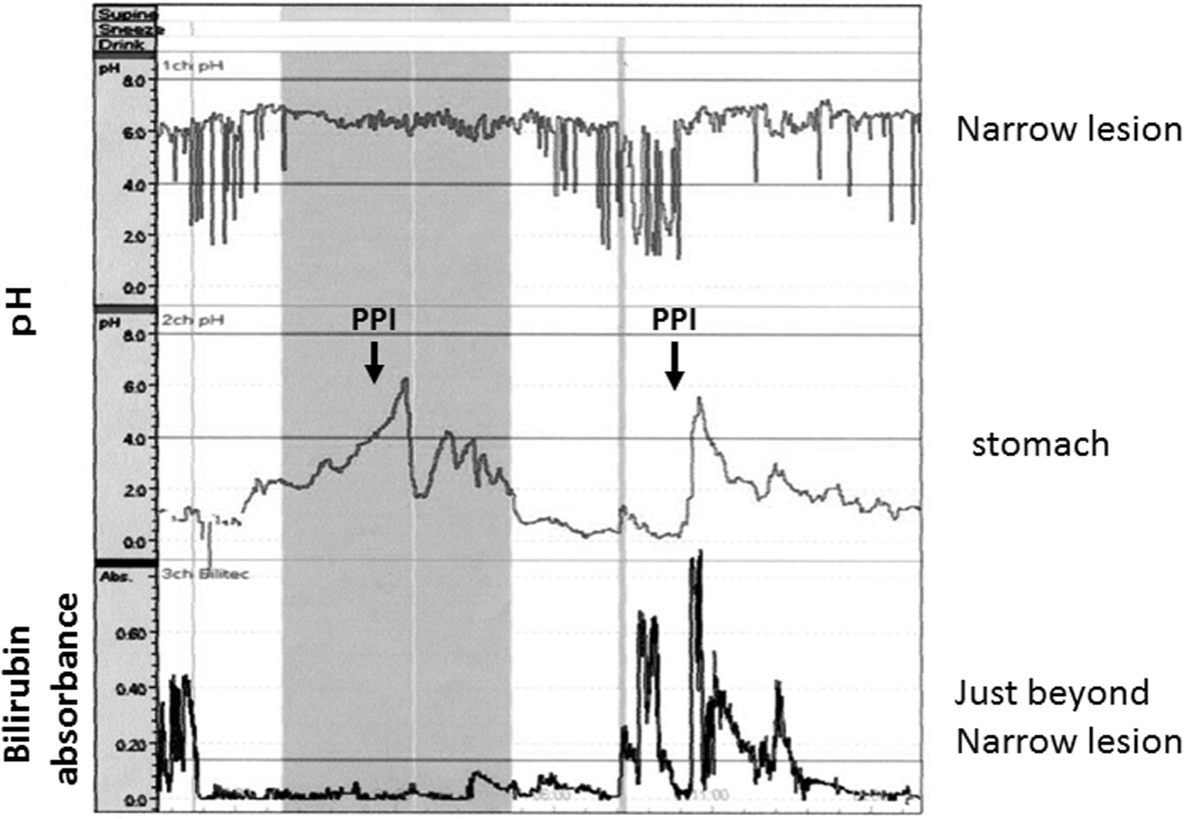
Simultaneous 24-h pH and bilirubin monitoring. In the narrow lesion, the proportion of time at pH < 4 was 3.0 %. With a drip of omeprazole of 20 mg, pH in the stomach rapidly increased to approximately 6 and but then rapidly decreased to less than 4. (Omeprazole treatment was started 3 days before the test.) The fraction of time with pH < 4 was 89.3 %. Just beyond the narrow lesion, the proportion of time at bilirubin absorbance >0.14 was 20.4 %

The upper gastrointestinal series revealed a long stricture extending from the middle esophagus to just above the cardia portion, the length of which was approximately 10 cm, and the sliding esophageal hiatal hernia (Fig. 1). Gastrointestinal endoscopy showed circumferential stricture of the middle esophagus with longitudinal esophageal ulcer scars (Fig. 2a). The narrow lesion was biopsied, and the result showed no malignancy. Preoperative gastrointestinal endoscopy before PpPD revealed a sliding esophageal hiatal hernia and mild esophagitis (Fig. 2b). We speculated that postoperative DGE, hiatal hernia, and gastric hyperacidity exacerbated the patient’s reflex esophagitis. The patient was treated with an H2 blocker for 2 weeks just after the surgery and with PPI from the 14th POD until the 140th POD. PPI was replaced to the H2 blocker due to the decreased numbers of white blood cells to less than 2000/μl from the 141th POD. The number of white blood cells recovered to normal level soon. Severe extensive stricture remained observed. We suspected that this patient had resistance to PPI; thus, we performed simultaneous 24-h pH and bilirubin monitoring to estimate the extent to which gastric acid secretion was inhibited by omeprazole (20 mg/drip/twice a day). Proximal and distal pH sensors were positioned in the narrow lesion and in the stomach, respectively, and a bilirubin sensor was positioned just beyond the narrow lesion. In the stomach, a pH < 4 was observed 89.3 % of the time (Fig. 3). Usually, in patients with GERD or intermediate and poor metabolizer CYP2C19 genotype, the proportion of time for which the stomach is characterized by pH < 4 decreases to approximately 50 % with PPI [4, 5]. In this study, after treatment with omeprazole for 6 days, the white blood cell count decreased from 4000 to 1700/μl; this level increased to normal levels soon after the course of medication had been completed. We recognized that this patient was resistant to PPI. Neither an H2 blocker nor PPI could prevent the exacerbation of reflux esophagitis. We therefore concluded that medication therapy could not suppress gastric acid and considered performing total gastrectomy to prevent gastric acid from injuring reconstructed organs and the remnant esophagus.

**Fig. 4:**
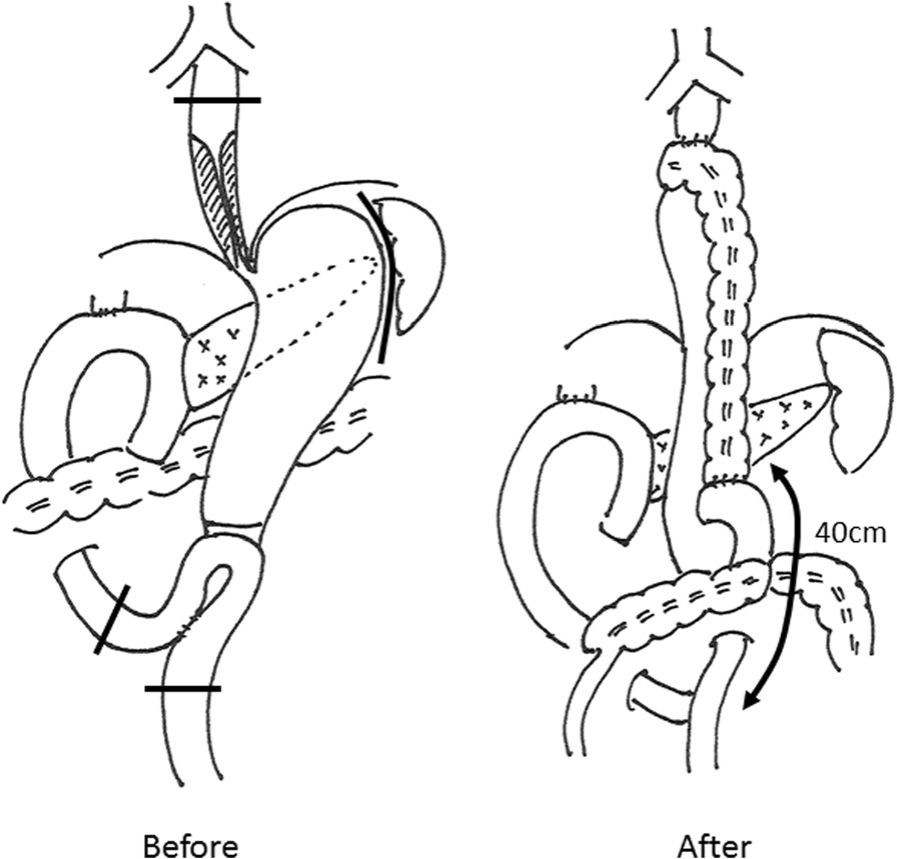
Schema of the operation. The first surgical procedure involved PpPD and Child reconstruction with Braun anastomosis. We performed middle-lower esophagectomy and total gastectomy through a right thoracoabdominal incision. The transverse colon with the vascular pedicle of the left colic vessel was pulled up to the cut end of the esophagus through a hiatus. The anal cut end of the transverse colon was anastomosed to the jejunum in a Roux-Y fashion. Jejunojejunostomy was performed 40 cm from the anal side of the colonojejunostomy

On the 158th POD, we performed resection of the middle-lower esophagus and total gastrectomy through a right thoracoabdominal approach. The middle-lower esophagus was hard; we therefore cut the esophagus just beyond the azygos arch. The length of the jejunum was not sufficient to pull up to the cut end of the esophagus due to Child reconstruction after PpPD, and we performed reconstruction using the transverse colon (Fig. 4).

The resected specimen showed wall thickening of the middle-lower esophagus, an ulcer scar on the 8-cm oral side of the cardia, an ulcer at the esophagogastric junction, and a petechial hemorrhage in the stomach. Pathological examination revealed no malignancy.

Minor leakage of the esophagocolonostomy developed postoperatively; the patient recovered rapidly under conservative treatment. He started oral intake on the 23rd POD and was discharged on the 34th POD in good condition. The CYP2C19 genotyping test performed after the second operation showed that the patient had a rapid metabolizer genotype. Two and a half years after the second surgery, gastroendoscopy and simultaneous 24-h pH and bilirubin monitoring were performed. There was no sign of either esophagitis or Barrett’s esophagus. Simultaneous 24-h pH and bilirubin monitoring revealed that the rate of fraction time bilirubin absorbance >0.14 was 0.7 % in the remnant esophagus; there was little bile reflux to the remnant esophagus. Nine years after surgery, the patient has good oral intake with good nutrition, without reflux esophagitis, symptom, or any evidence of recurrence. However, he has undergone gastrointestinal endoscopy once a year to detect early stage colon cancer which can be treated by endoscopic submucosal dissection in preventing surgical resection of reconstructed colon.

### Discussion

DGE is one of the major postoperative complications after PpPD, and the incidence has been reported to be 22–45 % [6]. The main pathogenesis of DGE after PpPD has been thought to be preservation of the pylorus ring without innervation. Conventional PD and subtotal stomach-preserving pancreatoduodenectomy (SSPPD), in which the pylorus and duodenum are removed and more than 90 % of the stomach is preserved, has recently been reported to reduce the incidence of DGE compared with PpPD [7–10]. In this patient, a sliding hernia was detected by preoperative gastroendoscopy. If DGE happened after surgery, substantial gastric acid reflux to the esophagus was expected to lead to severe esophagitis. Simultaneous 24-h pH and bilirubin monitoring revealed the reflux of not only gastric acid but also bile, resulting in severe esophagitis (Fig. 3). Conventional PD may reduce the risk of acid reflux because an antrectomy induces the reduction of the gastric acid secretion. Therefore, conventional PD or SSPPD with Roux-en Y anastomosis should have been selected to reduce the gastric acid secretion or the risk of DGE, and prevent bile reflux; otherwise, fundoplication such as Nissen and Toupet should have been added.

In this case, 24-h pH monitoring revealed that omeprazole could not sufficiently suppress gastric acid. The CYP2C19 genotyping test performed after the second operation showed that the patient had a rapid metabolizer genotype. Several studies have reported on the effects of CYP2C19 genotypic differences on PPI-mediated cure of GERD [11]. We suggest that the rapid metabolizer CYP2C19 genotype was one reason why extensive esophageal stricture happened so rapidly. In this patient, the decrease of white blood cell occurred due to the administration of PPI after the initial surgery and on the 24-h pH and bilirubin monitoring. We considered that PPI could not be administered after the second surgery due to the decrease in the white blood cell count caused by PPI. The results of simultaneous 24-h pH and bilirubin monitoring and the decrease of the white blood cell due to PPI could allow us to decide to perform total gastrectomy to protect the reconstructed organ and remnant esophagus from gastric acid. Simultaneous 24-h pH and bilirubin monitoring were very valuable in evaluating the pathogenesis of this case and choosing the operative procedure most likely to preserve the stomach.

Esophageal stricture of GERD is generally treated by endoscopic balloon dilation and continuous PPI administration; surgical resection is rarely performed due to the associated high morbidity and mortality [12]. Herein, we considered less invasive procedure such as balloon dilation or temporary stent replacement; however, because these treatments were expected to cause perforation again and because this patient had medication-resistant GERD, we decided to perform esophagectomy. There were some problems regarding surgical procedures, including the approach, the reconstructed organ, and the reconstructive route. We thought that mediastinitis due to perforation during balloon dilation might scar the tissue surrounding the esophagus and noted that it was difficult to separate the esophagus form adjacent tissue. As the transhiatal approach requires a blind separation maneuver, the right transthoracic approach was chosen. Esophageal stricture extended the middle esophagus, and the esophagus was cut just beyond the bifurcation of the trachea. Alimentary tract reconstruction was performed using the transverse colon (not the jejunum) because of the shortage of useful jejunum due to Child reconstruction after PpPD. With respect to the route of reconstruction, we elected to perform intrathoracic anastomosis because the patient refused the percutaneous route due to the poor cosmetic consequences. Roux-Y anastomosis was also added to prevent bile reflux to remnant esophagus. In fact, postoperative simultaneous 24-h pH and bilirubin monitoring revealed little bile reflux to the remnant esophagus.

## Conclusion

Long esophageal stricture after PpPD was successfully treated by middle-lower esophagectomy and total gastrectomy with transverse colon reconstruction through a right thoracoabdominal incision. Conventional PD or SSPPD with Roux-en Y reconstruction rather than PpPD should be selected to reduce the risk of DGE and prevent postoperative bile reflux, in performing PD for patients with hiatal hernia or rapid metabolizer CYP2C19 genotype; otherwise, fundoplication such as Nissen and Toupet should be added.

## Consent

Written informed consent was obtained from the patient for publication of this Case report and any accompanying images. A copy of the written consent is available for review by the Editor of this journal.

## Abbreviations

DGE: delayed gastric emptying
GERD: gastroesophageal reflux disease
POD: postoperative day
PPI: proton-pump inhibitor
PpPD: pylorus-preserving pancreatoduodenectomy
SSPPD: subtotal stomach-preserving pancreatoduodenectomy
